# Psychotic illness in people with Prader–Willi syndrome: a systematic review of clinical presentation, course and phenomenology

**DOI:** 10.1186/s13023-024-03026-y

**Published:** 2024-02-15

**Authors:** Lucie C. S. Aman, Suzannah D. Lester, Anthony J. Holland, Paul C. Fletcher

**Affiliations:** 1 Department of Psychiatry, University of Cambridge, Cambridge, UK; 2 Wellcome Trust MRC Institute of Metabolic Science, Biomedical Campus, Cambridge, UK; 3 Cambridgeshire and Peterborough National Health Service Foundation Trust, Cambridge, UK

**Keywords:** Prader–Willi syndrome, Psychosis, Cycloid psychosis, Atypical psychosis, Hallucination, Delusion, Genetic origin of psychosis, Early onset psychosis

## Abstract

**Background:**

Prader–Willi syndrome (PWS) is a rare and complex neurodevelopmental disorder resulting from absent paternal expression of maternally imprinted genes at chromosomal locus 15q11-13. This absence of expression occurs as a consequence of a deletion on the chromosome 15 of paternal origin (*ca.* 70%), a chromosome 15 maternal uniparental disomy (mUPD; *ca.* 25%), or an imprinting centre defect (IC; *ca*. 1–3%). At birth, individuals with PWS are severely hypotonic and fail to thrive. Hyperphagia and characteristic physical and neuropsychiatric phenotypes become apparent during childhood. The risk for the development of a co-morbid psychotic illness increases during the teenage years, specifically in those with PWS due to the presence of an mUPD. The primary aim of this literature review is to inform clinical practice. To achieve this, we have undertaken a systematic analysis of the clinical research literature on prevalence, presentation, course, characteristics, diagnosis and treatment of psychotic illness in people with PWS. The secondary aim is to identify clinical aspects of psychotic illness in PWS in need of further investigation.

**Methods and findings:**

A systematic literature review on psychosis in PWS was conducted on the databases Web of Knowledge, PubMed and Scopus, using the terms “((Prader–Willi syndrome) OR (Prader Willi Syndrome)) AND ((psychosis) OR (psychotic illness))”. All articles written in English and reporting original human research were reviewed. In all but three of the 16 cohort studies in which the genetic types were known, the authors reported higher rates of psychosis in people with PWS resulting from an mUPD, compared to those with the deletion subtype of PWS. When psychosis was present the presentation was psychosis similar regardless of genetic type and was usually characterised by an acute onset of hallucinations and delusions accompanied by confusion, anxiety and motor symptoms.

**Conclusions:**

The onset of confusion, an affective cyclical pattern with the presence of abnormal mental beliefs and experiences, usually of rapid onset is suggestive of the development of psychotic illness. Phenomenologically, this psychosis in people with PWS is atypical in comparison to schizophrenia and bipolar disorder in the general population. The relationship to psychosis in the general population and the optimum treatments remain uncertain.

## Prader–Willi syndrome and the association with psychotic illness

Prader–Willi syndrome (PWS) is a rare genetically determined neurodevelopmental disorder with a birth incidence of around 1:24,000 and a population prevalence of 1:45,000 to 1:52,000 [1]. As we describe below, high rates of severe psychotic illness, particularly in those with the rarer genetic form of PWS, have been reported and people with PWS may present to Accident and Emergency or mental health services serious ill and in crisis. The aim of this systematic clinical review is to evaluate existing clinical evidence so as to inform the identification, assessment and treatment of psychotic illness when it presents in people with this rare syndrome. This clinical review complements an earlier review that focussed on underlying mechanisms [2].

**Fig. 1:**
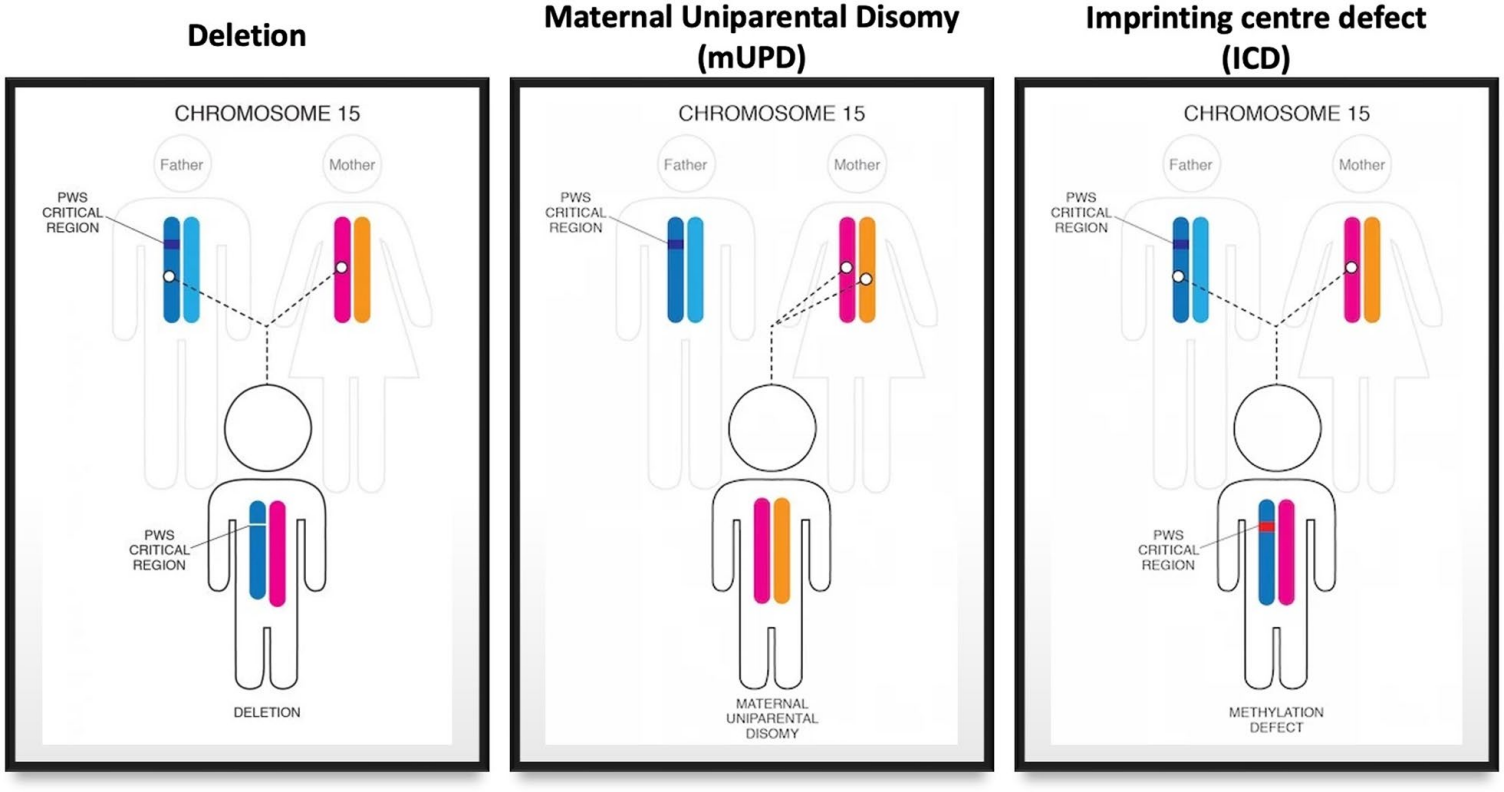
Genetic subtypes of Prader–Willi syndrome. Adapted from the foundation for Prader–Willi research (FPWR) website

PWS results from the absence or failure of expression of alleles of paternal origin of maternally imprinted genes located at the chromosomal locus 15q11-13. This arises as a consequence of one the following: 15q11-13 de novo deletion on the chromosome 15 of paternal origin (del; ca. 70%), chromosome 15 maternal uniparental disomy (mUPD; ca. 25%), an imprinting centre defect (ICD; ca. 3–5%), or an unbalanced translocation of chromosome 15 (< 5%) (see Fig. 1).

The main features of the syndrome at birth are extreme hypotonia and failure to thrive, followed by the development of hyperphagia in early childhood, which if not managed through controlling access to food, results in severe obesity and high rate of morbidity and mortality [3]. In addition, there is developmental delay associated with intellectual and social impairments, and short stature and impaired sexual development as a result of relative growth and sex hormone deficiencies, respectively. Specific neuropsychiatric symptomatology includes an increased propensity to severe emotional outbursts, anxiety, repetitive and ritualistic behavior, and severe skin picking [4]. For more detailed information about the manifestations of PWS, see [5, 6].

Whilst people with PWS, regardless of the genetic subtype, share a similar profile of non-psychotic psychopathology and problem behaviors, in the case of psychotic illness significantly higher rates are observedin those those with PWS due to (mUPD) compared with those with PWS due to paternal deletion. Given the rarity of the syndrome, and particularly of the rarity of people with the mUPD genetic type, few clinicians have experience in the recognition, diagnosis, and treatment of psychotic illness in this population. In the context of the broader complex neuropsychiatric phenotype characteristic of PWS, the challenge is to ensure that psychosis is diagnosed accurately and in a timely manner and that effective treatment and appropriate support is then provided.

## Methods

**Fig. 2:**
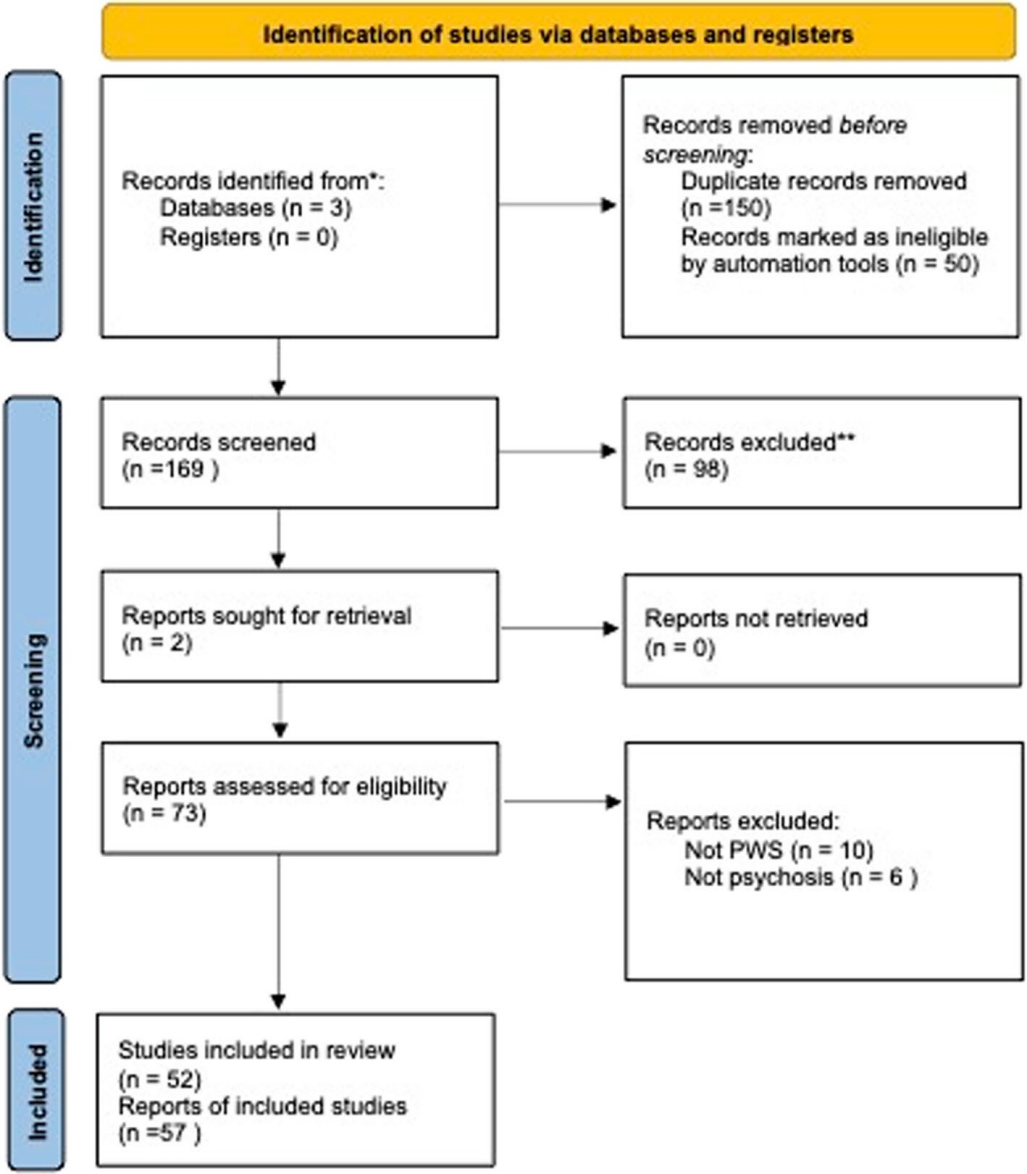
PRISMA flow diagram reporting the article identification process for systematic literature review of psychosis in Prader–Willi syndrome (PWS)

This systematic clinical review focuses specifically on psychotic illness in people with PWS and not on the broader PWS neuropsychiatric phenotype. The search was conducted on the databases Web of Knowledge, Pubmed and Scopus, using the terms “((Prader–Willi syndrome) OR (Prader Willi Syndrome)) AND ((psychosis) OR (psychotic illness))”. Automatic tools were used to reject articles not written in English, non-human research, and all reports that were not articles, case reports or reviews. All those articles selected were reviewed. Using these criteria, 169 original articles, published between 1987 and February 2023 were identified. Two additional articles, identified from article reference lists, were also included. The selection process is described in Fig. 2 using a PRISMA flow diagram [7]. A total of 73 articles were included for detailed analysis. Eighteen articles described cohort studies, and the overall number of individuals with PWS reported by these cohorts studies was 1556. Thirty-three articles were case reports, describing a total of 89 cases. In 58 of the 89 case reports, the PWS genetic subtype was reported (see Table 2), and in the other 31, genetic information was not available (see Table 3).

Literature searches were carried out between May 2019 and October 2023 by the first author.

The findings reported in the tables below are predominantly summaries of descriptive information about psychotic illness in this population, taken from both case reports and cohort studies. Using the more detailed and individual data from the 58 case studies in which genetic information was available, we also compared the presentation, course and phenomenology of psychotic illness in those who had PWS due to a mUPD and those who had a paternal chromosome 15 deletions and who also had had a psychotic illness. Because of similar genetic expression patterns, we group the very rare patients with imprinting centre defects with those with mUPD, and those with rare chromosomal translocations with patients with a deletion.

### Psychotic illness in PWS: an historical background

Prader et al. [8] first described what later became known as Prader–Willi syndrome in 1956. The occurrence of psychosis in someone with PWS was first reported 10 years later [9]. While several papers [10–13] noted a range of symptoms suggesting that the onset of psychotic illness was a specific feature of PWS, there was little reported in the literature on psychotic symptoms in people with PWS for two decades after these earlier reports. The symptoms of psychotic illness in PWS initially described in the above papers include references to hallucinations [14, 15] and paranoid delusion, agitation, and catatonia [10–13] (see Table 3). These papers were predominantly in the form of case studies and the findings could not be reliably generalized to the PWS population. However, these early cohort studies that focused particular attention on the presence of psychotic illness in PWS [11], along with two early cohort studies [16, 17] demonstrated clear evidence that specific phenomenology characteristic of psychotic illness was associated with PWS.

In the Beardsmore et al. [16] study, high rates of psychotic symptoms were reported in people with PWS compared to a control group of people with learning disabilities. Unfortunately, as genetic testing was not possible, the authors were unable to ascertain whether psychosis was linked to a specific genetic type of PWS. A later study by Clarke et al. [17] also confirmed the elevated occurrence of psychotic symptoms in people with PWS, with a prevalence rate of 6.3% in 93 people with a genetic diagnosis of PWS [17]. Notably, in this study, a high proportion of participants either had the deletion type (n = 34) or the genetic type was unknown (n = 55). Only four participants were confirmed as having an mUPD. The work described above established the link between PWS and psychosis, but the first definitive observation of a differential prevalence rate for psychosis according to genetic type of PWS did not appear until the beginning of this century.

### Differential rates of psychosis according to PWS genetic type

The first population-based study to indicate a difference in prevalence rates of psychosis in PWS according to genetic subtype reported only one out of 13 adults with deletion had had evidence of a psychotic illness, compared with five out of eight with mUPD [18]. Vogels et al. [19] also investigated psychotic illness in 37 people with PWS aged 13 years or older. Of the 37 individuals, 28 had deletion, and nine had mUPD. Of those nine individuals, six were found to have or have had a psychotic illness (none of the deletion participants were diagnosed with psychotic illness) [19]. In another study it was reported that of 16 people with PWS referred for neuropsychiatric evaluation because of relapsing psychotic illness, only one individual with PWS had the deletion subtype, while ten of the PWS individuals had mUPD,the genetic subtype of the remaining five was unknown [20].

**Fig. 3:**
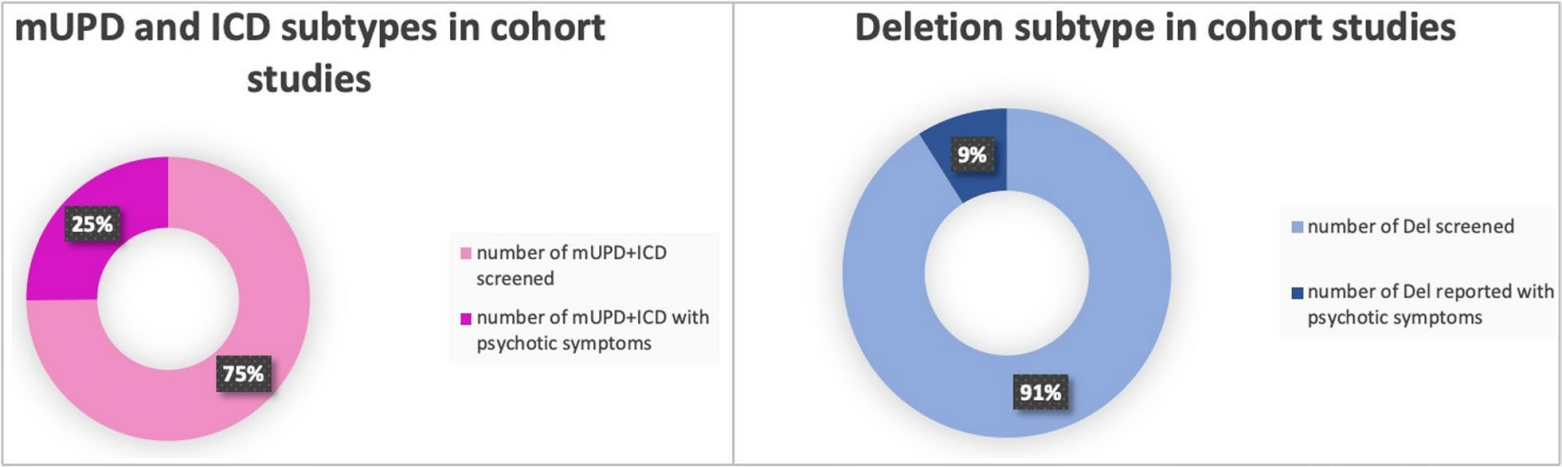
Proportion of reported psychotic symptoms in cohort studies according to PWS genetic subtypes

In more recent cohort studies, the reported prevalence of psychotic illness between the genetic subtypes of PWS varied. However, in most publications the prevalence of psychosis is higher in the mUPD group compared to the deletion group (see Table 1 and Fig. 3). In a cohort study, Soni et al. [28] estimated the incidences of psychiatric illness of 2.3 per 100 person-years in those with deletions and 6.7 per 100 person-years in those with PWS due to mUPD (see Table 1) [28].

**Table 1:**
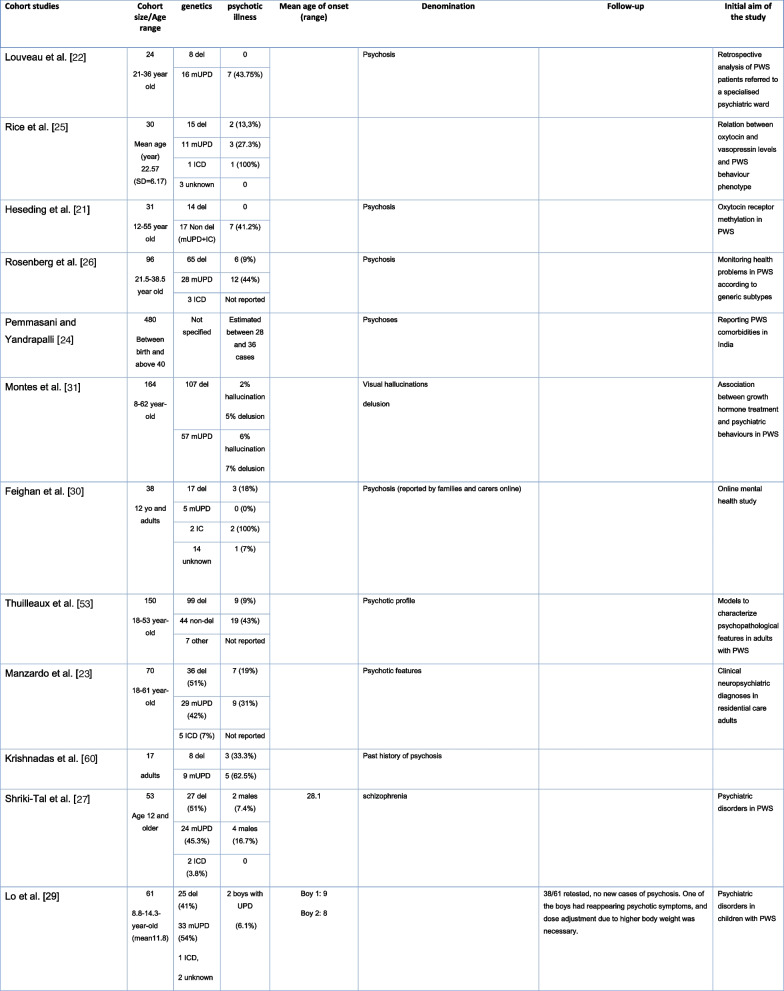

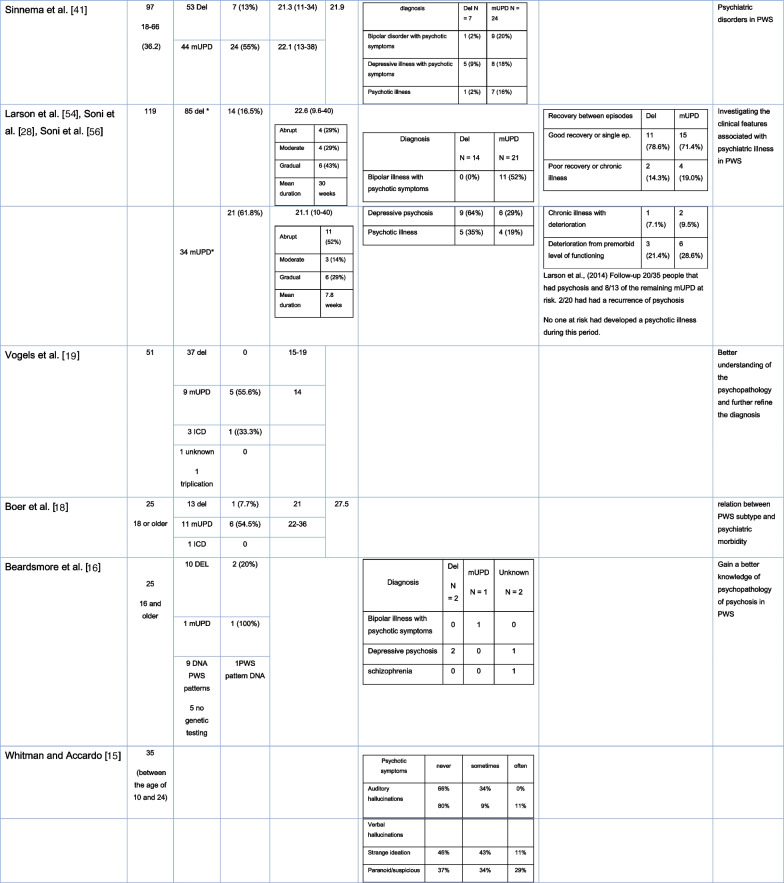
Cohort studies

*In this study, the author assimilated the individuals with an unbalanced translocation into the deletion group and those with ICD into the UPD group because of their respective genetical similarity

There are three cohort studies that did not find high rates of psychosis in individuals with the mUPD type. In a longitudinal study, 61 children with PWS between seven and 17 years old were assessed for various psychiatric disorders. Thirty-eight out of 61 were re-assessed two years later. During the first assessment, of the 34 individuals with mUPD, they found only two with psychotic disorder [29]. This discrepancy, when compared to other studies, may be due to the young age of the cohort. Most studies whose findings suggest higher rates of psychosis in mUPD, report an age of onset between 16 and 28 years [19]. A second study that did not find an association between psychosis and mUPD, was a national informant-based survey conducted in Ireland [30]. However, this lack of association might have occurred because 80% of individuals with the mUPD subtype were on antipsychotic medication. The third study to not find an association between psychosis and the mUPD type examined the association between growth hormone therapy (GHT) and psychiatric behaviours in PWS. One hundred and seven people with deletion and 57 with mUPD, aged between 8 and 62 years old, were studied to examine differences between genetic types, and differences between GHT and non-GHT participants. The study found no differential rate in psychosis between genetic types. However, limitations to the study’s findings include the fact that information on current and past psychiatric behaviors were collected using parent/guardian assessments, rather than the participants being assessed and diagnosed by mental health professionals. In addition, the participants’ mental health before enrolment in the study, as well as changes during the study, were not reported. No details were given of psychiatric medication use [31].

**Fig. 4:**
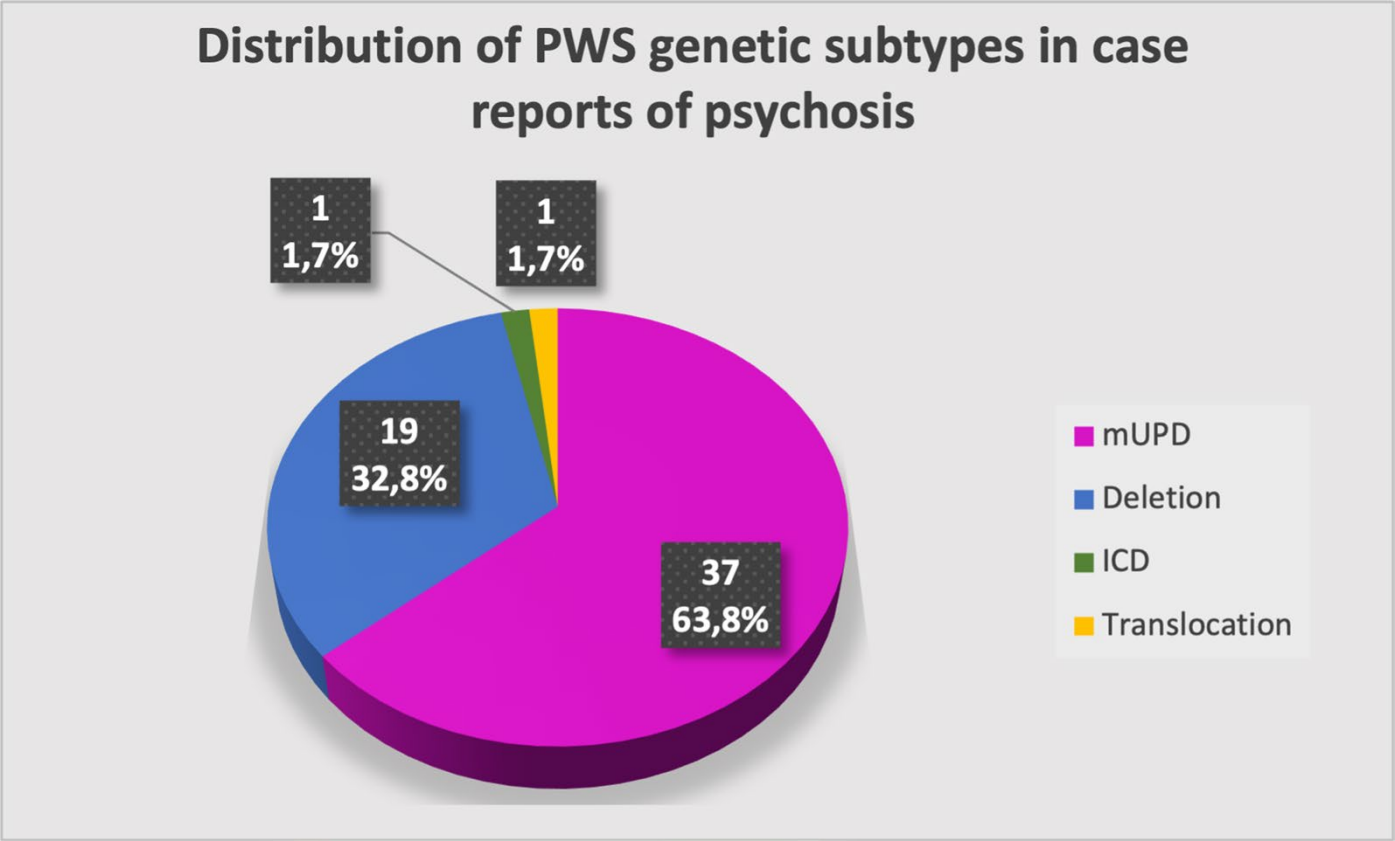
Rates of genetic subtypes in case reports of psychosis in PWS

Observations from the case reports support the association of psychotic illness with mUPD. As shown in Fig. 4 of the 58 people with PWS and psychosis described in the case reports who had their genetic type listed,, 37 (63.8%) had an mUPD, 19 (32.8%) had a deletion, one had an imprinting centre defect, and one had a translocation. This ratio of mUPD and deletion types is the opposite to what would be expected in relation with the known frequency of each genetic type. However, while the great majority of studies support the observation of an excess of psychotic illness in those with mUPD, it is also clear that people with PWS with a 15q11-13 deletions can develop such an illness, with rates in this population as high as 20%.

### Psychopathology

Table 1 summarizes the different psychiatric diagnoses made in the cohorts of people with PWS. In the early clinical research when there was limited agreement as to the main symptoms the classification of the psychotic illness associated with PWS was disputed. In the case studies listed in Tables 2 and 3 it is, however, possible to ascertain the phenomenology of the psychiatric illness in greater detail. The common features include hallucinations, persecutory delusions, heightened anxiety, motor symptoms (catatonia or agitation), confusional states, disturbed sleep (decreased or increased) and mood swings. The clinical picture is usually not consistent with accepted criteria for schizophrenia or specific psychotic disorders, and consequently the diagnosis made has often been atypical psychosis. Other diagnostic labels reported in the literature have, however, including schizophrenia and bipolar disorder with psychotic symptoms, and also cycloid psychosis, florid psychotic states, paranoic-hallucinatory psychosis, depressive psychosis and atypical bipolar disorder (e.g. [17, 20, 32–34]). At present there is no consensus as to the most appropriate diagnostic label for this atypical psychotic disorder. According to Verhoeven [35, 36], psychopathology, course, symptomatology and response to pharmacological treatment match with a diagnosis of cycloid psychosis, with the evidence from the clinical evaluation being closer to that of an atypical bipolar disorder [34–36].

**Table 2:** Case reports indicating the PWS genetic subtype

Cases reports with genetic subtype	Genetic subtype	Gender	Age at onset Type of onset	Symptoms	Other information	Outcome	Medication	Diagnosis
Singh et al. [37]	Del	M	16 sudden onsets	Acute psychosis, confusion, mood-incongruent delusions, hallucinations, anxiety, motility disturbances, mood swings	Growth hormone treatment	Complete remission of symptoms within a month	Not known	Cycloid psychosis Bipolar affective disorder
	mUPD	M	26, sudden onset	Acute psychosis, mood-incongruent delusions, anxiety, mood swings	Growth hormone treatment	Complete remission of symptoms within a month		Cycloid psychosis, Bipolar affective disorder
	mUPD	F	19, sudden onset	Acute psychosis, confusion, mood-incongruent delusions, hallucinations, anxiety, mood swings	Growth hormone treatment	Complete remission of symptoms within a month		Cycloid psychosis, Bipolar affective disorder
	mUPD	F	17, sudden onset	Acute psychosis, confusion, mood-incongruent delusions, hallucinations, anxiety, mood swings	Growth hormone treatment	Complete remission of symptoms within a month		Cycloid psychosis
	mUPD	M	26, sudden onset	Acute psychosis, mood-incongruent delusions, hallucinations, anxiety, mood swings		Complete remission of symptoms within a month		Cycloid psychosis
	mUPD	F	18, sudden onset	Acute psychosis, confusion, mood-incongruent delusions, anxiety, ecstasy motility disturbances, mood swings	Growth hormone treatment	Complete remission of symptoms within a month		Cycloid psychosis, Bipolar affective disorder
	mUPD	M	24, sudden onset	Acute psychosis, confusion, mood-incongruent delusions, hallucinations, anxiety, motility disturbances, mood swings	Growth hormone treatment	Mild auditory hallucinations despite resolution of other symptoms of cycloid psychosis		Cycloid psychosis, Bipolar affective disorder
	mUPD	F	22, sudden onset	Acute psychosis, confusion, mood-incongruent delusions, hallucinations, anxiety, ecstasy, motility disturbances, mood swings	Growth hormone treatment	Complete remission of symptoms within a month		Cycloid psychosis, Bipolar affective disorder
	Del	F	34, sudden onset	Acute psychosis, confusion, mood-incongruent delusions, hallucinations, anxiety, ecstasy, motility disturbances, mood swings		Complete remission of symptoms within a month		Cycloid psychosis, Bipolar affective disorder
	Del	F	31, sudden onset	Acute psychosis, confusion, mood-incongruent delusions, hallucinations, anxiety, motility disturbances, mood swings		Complete remission of symptoms within a month		Cycloid psychosis
	Del	F	31, sudden onset	Acute psychosis, confusion, mood-incongruent delusions, hallucinations, anxiety, motility disturbances, mood swings		Complete remission of symptoms within a month		Cycloid psychosis
Briegel [38]	mUPD	F	16, sudden	disorganized speech, reduced eye contact, signs of hallucinations, paranoid delusions, Aggressive behaviour, Increased agitation, refuse to eat	Bed wetting	Improvement 90% after 54 days of treatment	Aripiprazole Lorazepam	brief psychotic disorder
Kuppens et al. [39]	mUPD	M	Before 19 (recuurent episode at 19)			recocered	Valproic acid	
Poser et al. [40]	mUPD	F	25, within a month, 2 hospitalizations at 15 and 16 yo for depression with catatonia that responded to lorazepam and haloperidol	Manic behaviour increasing over a month: hyperverbal, euphoric, illogical**,** decreased sleep Then mute and withdrawn with catatonia	10 pounds weight loss in 10 days	Minimal improvement with pharmacological therapy Improvement after ECT and full recovery after 6 months	Aripiprazole**,** Lamotrigine Lithium**,** Lorazepam**,** Zisprasidone 8 electroconvulsive therapy	
Sinnema et al. [41]	mUPD	F	17	optic and acoustic hallucinations, delusions, aggression, motor agitation, increase of obsessive behaviour, increase of food obsession	bipolar	Stayed hospitalised 2 years, and had psychotic episodes all her further life	Anti-psychotic, Mood stabilizing agents, antidepressants	
Chiou and Tsai [42]	mUPD	M	15	Reference delusion, Physical aggression	Skin picking, ADHD	Great improvement after treatment	Risperidone 0.5 mg	
	Del	M	15, acute, medication-induced	Decreased need for sleep Physical violence	Autistic spectrum symptoms ADHD		carbamazepine	Medication-induced manic episode
Hergüner and Mukaddes [43]	mUPD	F	13, acute medication induced	Auditory and visual hallucination, paranoid delusions, social withdrawal, hypoactivity, echolalia, excessive sleep and decreased appetite		Symptoms completely resolved after discontinuation of fluoxetine		Medication induced psychotic symptoms
Verhoeven et al. [35], Verhoeven et al. [33], Verhoeven et al. [20]	mUPD	M	14 Within 2 weeks	Probable hallucinatory experiences, mood instability Increase in obsessional features		Stabilization of behaviour Marked effect of treatment	Thioridazine 200 mg with little effect, Lithium added	Cycloid psychosis
	mUPD	M	Within 2 weeks	Emotional turmoil, mood swings, anxieties Confusion		Marked and long-lasting effect of treatment. No occurrence of major behavioural problems over of a period of more than 2 years	Valproic acide 1500 mg	Cycloid psychosis but originaly diagnosed as bipolar affective disorder
	mUDP	M	Within 2 weeks	Emotional turmoil, mood swings, anxieties, confusion Paranoid ideations		Complete disappearance of psychotic symptoms within 6 weeks following start of lithium Stabilization and no new episode until the end of follow-up period (1 year)	Lithium	Cycloid psychosis
	Del	M	Within 2 weeks	Possible Perceptual disturbances, paranoid ideations, confusion Emotional turmoil, mood swings, anxieties Obsessive rituals		Moderate stabilization over a period of 12 months until the end of follow-up	Lithium Valproic acid carbamazepine	Cycloid psychosis
	mUPD	F	Subacute onset	Hallucinations; perceptual disturbances, Paranoid ideation Emotional turmoil; Mood swings, anxieties; confusion		Marked improvement	Lithium Valproic acid	Cycloid psychosis
	mUPD	F	Subacute onset	Hallucinations; paranoid ideation, emotional turmoil; Mood swings, anxieties; confusion Hyperactivity, Increased obsessive rituals		Marked improvement	Valproic acid	Cycloid psychosis
	mUPD	F	Subacute onset	Paranoid ideation, emotional turmoil; Mood swings, anxieties; confusion hyperactivity, increased obsessive rituals	hypothyroidism	Moderate improvement	Lithium Valproic acid	Cycloid psychosis
	mUPD	M	Subacute onset	Hallucinations, confusion, mood swings, increased obsessive rituals, paranoid ideation		Not evaluated	Valproic acid	Cycloid psychosis
	mUPD	F	Subacute onset	Emotional turmoil; Mood swings Paranoid ideation Confusion Anxieties; Hyperactivity, Increased obsessive rituals		Marked improvement	Lithium Valproic acid	Previous diagnosis: depression Last: cycloid psychosis
	mUPD	M	Subacute onset	Hallucinations, Probable perceptual disturbances and emotional turmoil, confusion, mood swings, anxieties, hyperactivity, increased obsessive rituals		Total recovery	Pipanperone	Previous diagnosis: delirious state Last: cycloid psychosis
Boer et al. [18]	Del	F	21				Antipsychotic, Mood stabiliser, antidepressant	paranoid delusion
	mUPD	M	28				Antipsychotic, Mood stabiliser antidepressant	Paranoid delusion and hallucinations
	mUPD	F	29				Antipsychotic	Paranoid delusion and hallucinations
	mUPD	F	22				Antidepressant	Paranoid delusion and hallucinations
	mUPD	F	36				Antipsychotic, Mood stabiliser, Antidepressant	Paranoid delusion and hallucinations
	mUPD	F	29				Antidepressant	Paranoid delusion and hallucinations
Vogels et al. [44], Vogels et al. [19]	ICD	M	15, Acute					
	mUPD	M	13, Acute					
	mUPD	F	19, Abrupt					
	mUPD	M	13, Acute					
	mUPD	F	16, Acute	Anxiety, agitation, restlessness, overactivity, confusion Aggressive behaviour, refused food and drinks Delusion	One of the episodes was preceded by fever and diarrhoeas during several weeks	After treatment with thioridazine, several mild psychotic episodes during the 13 next years. Then recurrent episodes again	Haloperidol, orphanedrine Thioridazine, Sodium valproate, risperidone, fluvoxamine	
	mUPD	M	19, Abrupt					
Beardsmore et al. [16]	mUPD	F	11, sudden	Food and drinks refusal, bedridden, refused human approach, Self-neglect		Relapsing episodes, Remission between episodes	Risperidone	Cycloid psychosis
	mUPD	M	13, sudden			Relapsing episodes, Remission between episodes	Risperidone	Cycloid psychosis
	Del	M	12, Not sudden	Agitation Mild anxiety, delusions, Hallucination		Remission with some residual symptoms	Risperidone	Transient psychotic disorder
	Del	M	14, Not sudden	Agitation, mild anxiety, delusions, hallucination		Remission with some residual symptoms	Risperidone	Transient psychotic disorder
Beardsmore et al. [16]	mUPD	F	< 33					Bipolar affective disorder with psychotic symptoms
	Del	F	23					Depressive episode with psychotic symptoms
	Del	M	33					Depressive episode with psychotic symptoms
Clarke [17]	Del	F	20	Delusion, paranoid ideation, unresponsive, withdrawn			thioridazine	Paranoid schizophrenia
	Trl	F	14	Disorientated, disordered speech, withdrawn, paranoid delusions		Several episodes. Good recovery between first episodes. Mental state stabilized following treatment with risperidone and carbamazepine	Trifluoperazine, Sulpiride, Risperidone Carbamazepine	Previous diagnosis: acute confusional state Cycloid psychosis
	mUPD	M		Aggression Delusion		Level of functioning has declined after illness	Chlorpromazine, Procyclidine Trifluoperazine	
	Del	M	12	Delusion, agitation, disturbed sleep, anxiety, apathy Refuse to eat and drink		Several psychotic episodes. Improvement at age 22 after treatment with haloperidol, sulpiride and fluoxetine	haloperidol, sulpiride and fluoxetine	Cycloid psychosis
Whittaker et al. [13]	mUPD	F	17	Querulous, tearful, anxious, withdrawn, paranoid thoughts Disrupted time orientation, sleep, appetite and concentration				
Watanabe et al. [45]	Del	F	19	anorexia, insomnia, guilt feelings, ideas of being doomed, ostracized and persecuted, and stupor alternating with agitation		Near-monthly rhythm, followed by a spontaneous remission in 7–18 days		Recurrent brief episodes Monthly rhythm
Ewald et al. [12]	Del	F	2 episodes of 3–4 weeks	Persecutory delusions, auditory hallucination, anxiety, psychomotor excitement				
Clarke [11]	Del	M	17, acute	Agitation, incoherent speech, labile emotions, hallucination (auditory and visual)		Completely resolved after 10 days. No recurrence after 3 years, even after chlorpromazine was discontinued	200 mg chlorpromazine	
	Del	F	24, acute	Agitation, anxiety, unable to sleep, refuse to eat, withdrawn	Temper outburst worst perimenstrually	Good recovery after 4 weeks but remain suspicious	Chlorpromazine, then dydrogesterine and fluphenazine	
	Del	F	acute	Anxious, restless Delusion, auditory hallucinations		discharged after 2 weeks but still abnormal beliefs. No return to premorbid level of functioning	thioridazine	
Bhate et al. [46]	Del	F		Restless both day and night, sleeping for only 1 or 2 h Episodes of psychosis with hallucinations and delusions	Frequent visit to the toilet, but incontinent

**Table 3:** Case reports for which the PWS genetic subtype is not available

Cases reports Without genetic subtype	gender	Age at onset Type of onset	Symptoms related to psychosis	Other symptoms	Outcome	Medication	Diagnosis
Sweeney et al. [47]	M	24	Agitation, paranoia then catatonia	Diabetes, hypotension, tachycardia		Lorazepam, bromocriptine and topiramate	Catatonia Following Cessation of Topiramate
Zwiebel et al. [48]	F	24	Catatonia	Hyponatremia hypoglyceamia	Remission of catatonia and return to baseline	lorazepam	Catatonia caused by oxcarbazepine withdrawal
Jacob et al. [49]	M	24	Pressure of speech, tangentiality, religious grandiose ideas, persecutory delusions, with ideas of reference congruent auditory hallucinations		Became more settled and mood became euthymic	Risperidone Semisodium valproate (mood stabilizer)	manic episode with psychotic symptoms bipolar affective disorder
	F	28	Agitation, verbal and physical violence, withdrawn, auditory hallucination	Wetting bed	Dramatic improvement on risperidone but developed hyperprolactinemia. Responded well to quietiapine	Fluoxetine (antidepressant) Risperidone, then quetiapine	Psychotic illness
	M	31	Low mood, delusions, hallucinations, inappropriate behaviour		First episode responded well after 3 weeks of treatment	Citalopram (antidepressant), Risperidone, Mirtazapine	
Verhoeven et al. [35], Verhoeven et al. [33] Verhoeven et al. [20]	M	Within 2 weeks	Hallucinations, perceptual disturbances Emotional turmoil, anxieties, confusion Mood swings, Sleep disturbance		Psychotic symptoms disappeared after 4 weeks, improved behaviour, increased concentration, and social skills. Marked effect of treatment	Lithium Haloperidol 2–4 mg	acute polymorphic psychotic disorder
	M	16 Within 2 weeks	Visual and auditory hallucinations, perceptual disturbances Paranoid ideations, confusion, psychomotor agitation Emotional turmoil, mood swings, anxieties, sleep disturbance		Disappearance of all psychotic symptoms within a few weeks. Stabilization of motor activity, sleep rhythm, and mood levels. At follow-up 9 months later, functioning at pre-morbid level	Lithium Haloperidol 3.5 mg	Previous diagnosis: bipolar disorder; Transient psychotic disorder Last diagnosis: acute polymorphic psychotic disorder
	F	Subacute onset	Hallucinations; perceptual disturbances paranoid ideation Emotional turmoil; Mood swings, anxieties; confusion Hyperactivity, Increased obsessive rituals		Marked improvement	Valproic acid	Cycloid psychosis
	M	Subacute onset	Hallucinations; paranoid ideation, Emotional turmoil; Mood swings, anxieties; confusion Hyperactivity, Increased obsessive rituals		Marked improvement	Valproic acid	Cycloid psychosis
	M	Subacute onset	Hallucinations; paranoid ideation, Emotional turmoil; Mood swings, anxieties; confusion Hyperactivity, Increased obsessive rituals		Marked improvement	Valproic acid	Cycloid psychosis
	F	Subacute onset	Anxieties Hyperactivity, Mood swings, Increased obsessive rituals, Paranoid ideation	ACTH deficiency, narcolepsy		Valproic acid	Cycloid psychosis
	M	Subacute onset	Hallucinations; perceptual disturbances paranoid ideation, Emotional turmoil; Mood swings, anxieties; confusion Hyperactivity, Increased obsessive rituals		Marked improvement	Valproic acid Haloperidol 2 mg	Previous diagnosis: paranoid psychosis Last: cycloid psychosis
Beardsmore et al. [16]*	F	29					Depressive episode with psychotic symptoms
	M	27					Schizophrenia or delusional disorder
Clarke [17]*	F	21	Hallucinations, reduced appetite, anxiety			Paroxetine Trifluoperazine	Cycloid psychosis
	F	16	Auditory hallucinations, anxiety, agitation, aggressive behaviour Refuse drinks and food, social withdrawal, sleep disturbance		Good recovery between episodes	Haloperidol	Cycloid psychosis
Takhar and Malla [50]		Mid 30 s	Delusions, hallucinations, confusion Moderate disorganization in thinking	Multiple gastro-intestinal problems, urinary infections and incontinence, hepatitis, hiatal hernia, hypothyroidism	Fluphenazine during 15 years induced a parkinsonian syndrome. Clozapine prescribed instead	Fluphenazine then Clozapine	
Tyndall and Fitzpatrick [51]	F	15, acute	Possible paranoid delusions and visual hallucinations developmental regression, tearfulness, agitation, sleep disturbance		Rapid and complete remission	No response to amitriptyline. Oral Flupenthixol	
Clarke et al. [10]	F	20	Increasingly suspicious, auditory hallucination probable		Return to premorbid behaviour	Flupenthixol	Delusional disorder
Bartolucci and Younger [52]	F	22, Sudden	1st episode: uncommunicative, Anxiety, Insomnia 2nd episode: refuse to eat and drink Possible auditory and visual hallucination		Completely resolved first episode 8 years later new episode No lasting improvement	1st episode: chlorpromazine 2nd episode: tricyclic anti-depressant, fluoxetine with improvement. Lithium with no change. Lorazepam and chlorpromazine, haloperidol, disodium valproate buspirone	
	M	13	Auditory hallucination				
	M	21	Auditory and visual hallucinations				
	M	22					
	M	27	Auditory and visual hallucination				
	F	28					
	F	30					
	M	30					
	F	33	Possible auditory and visual hallucinations				
	M	34					
Bray et al. [14]			Severe withdrawal from interpersonal interaction, regressive behaviour		Spontaneous remission		

First patient was non-deletion, second had a clinical diagnosis

**In cases 1 to 9, 7 had a deletion and 2 individuals had only a clinical diagnosis of PWS

Other authors have classified the psychotic symptoms in PWS more generally. In 1994, Bartolucci and Younger [52] reported nine cases of neuropsychiatric disorders in people with PWS. They were slightly different in characteristics from known psychotic disorders in the typically developing population with, in general, an earlier onset, cycloid patterns and atypical presentation. They classified these psychiatric symptoms as: (1) trait fluctuation (changes in behavioral and vegetative traits such as refusal of food and drink, food binges, escalation of antisocial behaviour); (2) lethargic-refusal states (refusing the approach of others, having no energy and being in a state of self-neglect); and (3) psychotic states (agitation, insomnia, decrease in hyperphagia, attentional deficits) [52]. More recently, Thuilleaux et al. [53] proposed a model to characterise psychopathological features common in adults with PWS. This model includes a profile characterised by ‘a psychotic disorganization of ideas, emotions, and behaviour that is persistently present as a personality trait’. The profile they describe includes loss of links with reality, with or without hallucinations; delusional ideation; strange and disorganized behaviour; and negative symptoms [53]. It is important to acknowledge that both of these studies published some years apart took a very broad view of psychosis, including in their overall descriptions non-specific symptoms, such as sleep and eating behavior changes. It is difficult to interpret their findings precisely or to relate them specifically to the presence or not of psychosis.

**Fig. 5:**
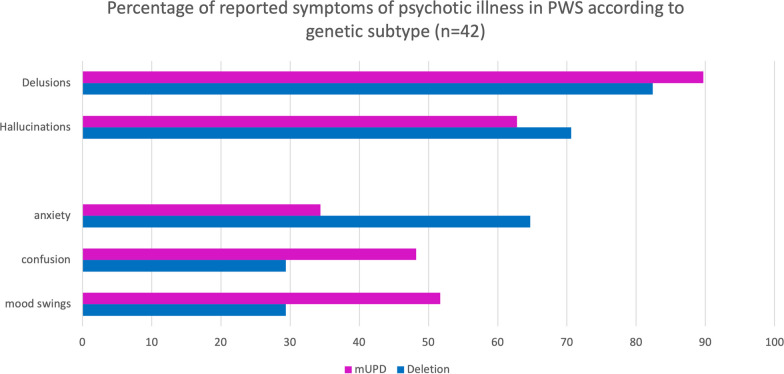
Percentage of psychiatric symptoms according to genetic subtype reported in case study reports

Soni et al. [28] compared the phenomenology of psychosis in people with PWS who had a mUPD with the cases of psychosis in people with PWS due to a deletion. The question they were seeking to answer was whether a psychotic illness, if it was to develop, was the same in people with PWS regardless of genetic type. She reported more severe affective co-morbidity in those with a psychotic illness and mUPD, but with broad similarities in phenomenological findings and the diagnostic category of affective disorder, except for the duration of the first psychotic episode. Analysis of the 56 case reports included in this review, also found similar levels of positive symptoms, such as delusions and hallucinations, but in contrast to Soni et al. [28] there were higher levels of anxiety in those with a paternal deletion, and more confusion and mood swings in those with mUPD (see Fig. 5).

### Age of onset

The age of onset of psychotic illness in people with PWS is highly variable. In the reviewed articles, where it was specified, we found an age of onset ranging between eight years [29] and 40 years of age [28]. The age of high risk is generally during the teenage years or early adulthood, and tends, on average, to be earlier than the onset of psychotic illnesses in the general population. (See Table 1 for mean age of onset in cohort studies.). The mean age of onset differs between studies largely because of the relatively small size of most of the samples and differences in the way in which age of onset was established.

### Type of onset

Most case studies report an acute onset [17, 20], whereas longitudinal studies report various types of onsets. Vogels et al. [19] in their study including 59 people with PWS followed up for a minimum of 10 years, described six people with psychotic symptoms, all with acute onset. However, Soni et al. [28] found in their cohort study only a slightly increased likelihood of acute rather than insidious onset.

### Duration

The duration of first episodes of psychosis varied. According to Soni et al. [28] those with mUPD experienced a shorter duration of a first major psychotic episode compared to first episodes of psychosis in individuals with deletion genetic type, with a good recovery between episodes and overall, a good response to treatments. In a follow-up study, Larson et al. [54] identified 20 people with PWS due to mUPD who had had a psychotic illness and had been seen as part of the Soni et al. [28] study [28, 54]. Two or more years later, two of the participants had a relapse of their psychotic illness. The others remained mentally well but on psychiatric medications (see Table 1 for more details).

### Risk and protective factors

Some case and cohort studies have reported details of the circumstances in which the first episode, and sometimes the subsequent episodes, occurred. When reported, the first episodes often indicated the presence of a potential stressor, such as a life event, a physical illness or a change in routine and/or environment (e.g. severe illness or death of a relative, change of school or diet, holidays) [17, 38, 44]. It has been proposed that developmental brain changes associated with adolescence and an underlying genetic predisposition to affective disorder and psychosis associated with PWS results in an increasing vulnerability to psychosis with age. The illness first becomes apparent in adolescence or early adult life as a consequence of some additional environmental stressor (see [55]). What is uncertain is whether treatments, such as growth hormone and various psychiatric medications, when administered from childhood reduce the risk of the subsequent development of psychotic symptoms. Interestingly, Background genetics may also be important particularly in those with a deletion. Soni et al. [28] reported that 50% of the deletion group with a history of psychosis in her cohort also had a history of an affective disorder in a first-degree relative, and in all cases it was in the mother. In some cases the affective disorder had been present before the child with PWS was born suggesting that maternal depression could not in these cases be explained by the emotional impact of having a child with PWS. This led to the authors proposing a two hit model for psychotic illness: the first is a predisposition to affective instability common to PWS regardless of the genetic type. The second hit is associated with the specific psychopathological effects of having an mUPD or, in the case of those with a deletion, a genetic loading for affective disorder on the maternal side.

Soni et al. [56] noticed that life events preceding a first episode of psychosis were often associated with physical illness in addition to changes in routine. The hypothesis that an immune response might trigger the onset of a psychotic illness is supported by reports of some individuals with PWS developing psychosis alongside physical symptoms. Vogels et al. [19] reported that all six patients with PWS and psychotic symptoms described gastro-intestinal problems in addition to other physical symptoms, such as fever and sore throat. Similarly, Takhar and Malla [50] described a person with PWS with multiple gastro-intestinal problems, urinary incontinence, urinary infection and hepatitis. Some limited support for an immunological component comes from a recent study of 20 participants with PWS. The authors reported that higher levels of IL-1β in participants with PWS were associated with more severe symptoms of withdrawal/depression and thought problems. These symptoms are associated with higher risk of developing psychotic and bipolar disorders [57].

Given the reported presence of confusion and the possibility of a physical illness triggering an immune response, the possibility of an auto-antibody response to specific neural tissue as the cause of the symptoms needs investigation. However, why this would cause the psychosis to predominately affect those with PWS due to mUPD is unclear. Alternatively, the urinary and gastro-intestinal problems might be explained by an autonomic nervous system dysfunction. All of the six people with PWS who developed psychosis in the Vogels et al. [44] study showed gastro-intestinal symptoms, and two had enuresis. Bhate et al. [46] reported psychotic symptoms and enuresis (with no obvious cause) in a 36-year-old women with PWS. Autonomic system dysfunction is associated with an increased risk of psychosis in the general population; and heart rate variability is known to be reduced in people with schizophrenia and individuals at high risk of psychosis [58].

Modifications in the dosage of medications whose actions are on the brain have also been reported to precede the onset of psychosis. One case of rapid-cycling bipolar illness and one case of psychotic illness were thought to have been triggered by the sudden withdrawing of the appetite suppressants, fenfluramine and sibutramine. Fenfluramine is known to increase serotonin availability in the brain and sibutramine inhibits re-uptake of serotonin, noradrenaline and dopamine [56]. Similarly, Herguner et al. described a 13-year-old girl with PWS whose psychosis had apparently been triggered by the administration of the selective serotonin reuptake inhibitor, fluoxetine [43]. Frances et al. [59] also indicate that antidepressant drugs may have precipitated mania or rapid-cycling bipolar disorder. A 2018 neuroimaging study found that participants in the mUPD group had lower brain-stem serotonin receptor availability compared with the deletion group. This might signify that those with mUPD have a lower synaptic serotonin concentration compared with those with deletion, and might be an indicator as to why there is the greater prevalence of affective psychotic illness in this specific population [60].

Whether changes in sex hormone levels with age or the atypical sex hormone environment consequent upon impaired hypothalamic function associated with PWS is relevant to the onset of psychosis is unknown. However, Bhate et al. [46] described a 36-year-old woman with PWS who developed psychosis a few months after the onset of secondary amenorrhea and one patient, described by Clarke et al. [17], developed psychotic symptoms after a second injection of testosterone. Soni et al. [28] observed that some features of psychosis in PWS are similar to those that develop during post-partum psychosis (bipolarity and confusional states), an illness that occurs at a time of hormonal change.

It has been reported that growth hormone could have a protective action, limiting the risk of developing psychosis by improving cell-to-cell communication in the brain [61]. However, Singh et al. [37] reported that seven out of 11 cases of people with PWS who had been treated with growth hormone also developed a co-morbid psychotic illness. Given that a generation of children treated with growth hormone during early childhood are now reaching the age of risk of developing psychosis, further cohort studies are required to investigate the effects of growth hormone therapy on the risk of psychopathology in people with PWS [37].

### Treatment

There have been no controlled trials that have specifically investigated the outcomes of treatments for psychotic illness when using established psychiatric medications in people with PWS. The quality of the evidence on treatment is therefore at the level of clinical opinion. The general approach in clinical practice has been to use atypical neuroleptics, anti-depressants and mood stabilising medications, as is standard in the general population. When the names of medications were included in the paper, they have been listed in Tables 2 and 3. However, it is difficult to draw any conclusion regarding treatment because information is rarely given in sufficient detail. Moreover, very little data is available on long-term mental state stability following specific treatments. We found only three cohort follow-up studies in this literature rsearch. Larson et al. [54] reported that of the13 out of 22 people who had been psychotic, the majority had done well but only two had been taken off their anti-psychotic medication after two years. Soni et al. [56] followed up the participants in their cohort 2.5 years after the original study. Individuals with mUPD were more likely to experience recurrent episodes, and usually had been prescribed a greater number of psychiatric medications before finding one that elicited a satisfactory response. In this study, the authors also state that once the right medication is found, individuals with PWS and psychotic illness had a sustained positive response to antipsychotic and antidepressant drugs in general, and that patients treated with these medications are less likely to develop an episode of illness in the future [56].

### Psychopharmacology

Based on the case reports it seems that risperidone, an atypical anti-psychotic, is often used with a satisfactory outcome. This is confirmed by a study published by Bonnot et al. [62]. However, a clinical trial on pharmacological treatment of psychosis in PWS is needed. The risks and benefits of using antidepressant medication in people with PWS experiencing or at risk of psychosis is still uncertain. Soni et al. [56] reported that fluoxetine and other SSRIs, were commonly prescribed. However, Frances et al. [59] indicated that antidepressant drugs may precipitate mania or rapid-cycling bipolar disorder. A case report published in 2007 also described a 13 year-old girl with PWS who had psychotic symptoms triggered by the administration of fluoxetine [43]. Mood stabilizing medications, such as lithium, have also been used in combination with anti-psychotic drugs, therefore making it difficult to assess the effectiveness or each. Many studies described patients taking mood stabilising medications, but very few studies reported if the medication had been started before, with or after anti-psychotic medication, and the outcomes are rarely reported. Verhoeven et al. [35] described positive effects of mood stabilizing medication on the course of illness and preventing relapses, but this was not found by Bartolucci and Younger [52] and Soni et al. [56].

## Conclusions

The rarity of PWS and particularly of those with the mUPD genetic subtypes makes systematic studies of adequate size problematic and the evidence limited. In addition, most psychiatrists are unlikely to see many affected people, if any, and therefore their experience will be limited when it comes to assessment and treatment. The aim of this review is to try to remedy this by bringing the available evidence together to inform clinical practice.

Given the nature of the existing literature, we carried out a primarily descriptive analysis based on information gathered from case reports and cohort studies. While some cohort studies allowed the differential risk for psychotic illness, depending on the genetic type, to be analysed, in many instances they did not provide specific descriptions of psychotic symptoms. Furthermore, in some earlier studies, genetic information was either not present or incomplete.

The evidence reviewed in this paper from several sources indicates a high risk of psychotic illness among those with the mUPD subtype. This risk is observed to increase during the teenage years and early adulthood and is seen to plateau in adult life. In cohort studies, the rates of psychosis in those with mUPD are significantly greater than for those with the deletion subtype, with prevalence rates of psychosis in those with mUPD as high as 60%. However, it is important to note that when case and cohort studies are combined, the evidence suggests that psychotic illness in people with PWS due to a mUPD is not inevitable.

### Nosology

The precise diagnostic status of the psychotic illnesses remains unclear. The studies suggest a strong affective component with mood instability and hypomanic and depressive phases and the presence of delusions and/or hallucinations. Strikingly, confusion has been described as a feature, although it is uncertain whether that includes disorientation in time and place. What is described as “confusion” may be similar to what might be best described as perplexity. Motor abnormalities have also been reported. This combination is similar to the profile of symptoms for the diagnosis of cycloid psychosis [63]. Their overview of cycloid psychosis provides a useful historical background summarizing the original work of Leonhard, who argued against Kraepelin’s schizophrenia and manic-depressive illness divide and making the case that cycloid psychoses (in plural) as a third group of psychotic illnesses. This third group has beendescribed as a delusional affective psychosis or ‘anxiety-elation’ psychosis and conceptualised according to Leonhard as a combination of symptomatology across bipolar axial syndromes: anxiety-happiness, excitation-inhibition-confusion, and hypermobility-hypomobility. Later work introduced operational criteria [64] with the main defining features including acute onset, remitting course, symptom polymorphism, and benign outcome in the long term [63]. In the DSM and ICD systems, cycloid psychosis has not been included. From a nosological perspective, the clinical picture of psychosis affecting people with PWS is certainly close to that of cycloid psychosis and appears to be closer to an affective disorder than to schizophrenia.

The presence of psychosis should therefore be considered where there is a clear and sudden deterioration in behavior and mental state, perhaps preceded by a physical illness or a stressful life event.

Importantly, the onset, mental state characteristics and persistence over time distinguish it from other manifestations of the wider PWS neuropsychiatric phenotype, such as severe behavoral/emotional outbursts. Such outbursts are common among people with PWS and are often present since childhood and triggered by change or frustration. These are not by themselves indicative of psychotic illness (see [6] for a detailed description of the neuropsychiatric phenotype).

### Treatment

In the absence of any formal treatment trial for psychotic illness in people with PWS, the assumption has been in the literature that standard treatments for psychosis and/or affective disorders are appropriate. One outcome study suggests a good prognosis with such an approach but with the probable need for continued psychiatric medication [54]. As described in this review, the use of multiple medications, given at different times in the course of the illness and at different doses, limits the conclusions that can be drawn from the papers reviewed. The evidence summarized in this review indicates that the onset is acute and can be severe, and therefore initial treatment, once a diagnosis has been established, will almost certainly be the use of antipsychotic or sedative medication to reduce agitation and to treat any mood disorder and abnormal mental beliefs or experiences. Clinical practice, when treating mental illness in people with a neurodevelopmental disability, is to start at a lower-than-normal dose and increase slowly, depending on clinical response. For people with PWS, an added problem is the effect of psychiatric medication on appetite and weight and for this reason olanzapine is best avoided. Additional co-morbidities that may be weight-related, such as sleep apnea, are also of concern. However, the problem remains that the level of evidence is limited. Multi-centred longitudinal studies, which are able to recruit sufficient numbers of people with PWS of both genetic types reaching the age at risk for psychotic illness, are needed. Such a clinical study could focus on the identification of early clinical and other risk markers for developing psychosis and identifying life events and antecedents that precede psychosis to inform our understanding of aetiology.

### Future clinical research

A multi-site study might also identify sufficient numbers of people with mUPD who are mentally stable or have passed the age most at risk of psychosis to provide clues as to potentially individual or environmental protective factors, both at the level of the individual and the environment. The question remains as to whether the aetiology and underlying mechanisms that result in the development of a psychotic illness are the same in those with a deletion compared to those with mUPD. Clinically, the psychotic illness seems similar but not identical, with more affective symptoms in those who have a mUPD. In addition, Soni et al. [28] suggests maternal history of affective disorder as having a potential role in the development of psychosis in those with a deletion who develop psychotic symptoms, suggesting further investigation of maternal affective disorder is warranted. Furthermore, this observation of a differential risk for psychotic illness, dependent on genetic type, points to potential novel genetic mechanisms that may inform our understanding of psychotic illness in the general population. Our work on these possible mechanisms has been published in [2].

### Service implications

While not specifically covered in this review, what follows from the findings is the need for services that are able to respond rapidly when the behavior of someone with PWS deteriorates and when a psychotic illness is suspected. A clear diagnostic and treatment pathway should be available in all localities with the necessary expertise to make the diagnosis, particularly distinguishing psychotic illness from the wider PWS neuropsychiatric phenotype, and thereby offering the correct treatment. From the description in some case reports and cohort studies it is also clear that either highly staff-resourced community-based first episode psychosis services or short term psychiatric in-patient care may be needed, with the additional requirement that such environments must be able to manage the food environment. Particularly with the potential effects of psychiatric medications on the drive to eat and subsequent increased weight, the absence of food security would have a serious deleterious effect on physical health.

**Table:** 

**Key information about psychosis in Prader Willi Syndrome** ♦ Prevalence: • High prevalence of psychosis in mUPD, but also present in delPWS ♦ Onset: • More likely to be acute onset, at least in mUPD • Has been reported as early as 8 years old • Usually during late adolescence or early twenties • The onset can also occur in adult life ♦ Psychopathology: • Anxiety, hallucinations and delusions are the most commonly reported symptoms • Confusional state and motor symptoms often present (catatonia or agitation) • Most common diagnosis is atypical psychosis, often with cycloid or bipolar components ♦ Treatments: • Treatment with Risperidone seems promising • Overall a good recovery between episodes	

**Table:** 

**Future research** ♦ Investigate currently available treatment efficacy and outcome in PWS, especially Risperidone ♦ Determine the effect of growth hormone treatment on psychopathology in PWS ♦ Investigate potential mechanisms and precipitants • Microbiome • Immune system • Autonomic system • Systematically note potential precipitant events	

## Data Availability

All data generated or analysed during this study are included in this published article. All data supporting the findings of this study are available within the paper, and more specifically within the tables. All articles on psychosis in PWS have been included in the review, and listed in the tables. Their reference can be found in the bibliography section.
